# Comparative Analysis of Mean Pleural Fluid D-dimer Level in Malignant and Non-Malignant Pleural Effusion Patients

**Published:** 2019-01

**Authors:** Mohammad Emami Ardestani, Mohammad Modaemzadeh, Ali Reza Mohammadi

**Affiliations:** 1 Departments of Pulmonology, Internal Medicine, School of Medicine, Isfahan University of Medical Sciences, Isfahan, Iran; 2 Internal Medicine Department, Isfahan University of Medical Sciences, Isfahan, Iran; 3 Isfahan University of Medical Sciences, Isfahan, Iran

**Keywords:** Pleural effusion, Malignancy, D-dimer

## Abstract

**Background:**

Malignant Pleural Effusion (MPE) is a condition that mostly presents with dyspnea. There are some ways to distinguish it from Non-Malignant Pleural Effusion (NMPE).The aim of this study was to compare serum and pleural D-dimer levels between MPE and NMPE patients.

**Materials and Methods:**

Patients diagnosed with Pleural Effusion (PE) were selected to participate in this study. They were allocated in 2 groups of MPE and NMPE according to the etiology. Serum and pleural fluid D-dimer level were measured and statistically analyzed between two groups.

**Results:**

32 MPE patients and 32 NMPE patients participated in this study. The mean age was 61.3 ± 12 years and M/F ratio was 35/29. The mean pleural and serum D-dimer levels were 3472± 1312 ng/dl and 3259±1220 ng/dl in patients with MPE, and 3425 ± 32.5ng/dl and 2425 ± 1311ng/dl in patients with NMPE, respectively. The serum D-dimer levels were not statistically different between 2 groups; while the pleural D-dimer levels were higher in MPE group in comparison with NMP patients (P<0.05).

**Conclusion:**

This study showed that pleural D-dimer levels were significantly different between two groups and therefore pleural D-dimer can be considered as a non-invasive tool for diagnosis of MPE.

## INTRODUCTION

Pleural Effusion (PE) is defined as collection of fluid in pleural space and classified into two categories of exudative and transudative PE (1). PE is the result of a wide range of medical conditions including infection, malignancy, trauma, collagen vascular disease, etc. (2,3). Malignancy is one of the main causes of exudative PE, which is called Malignant Pleural Effusion (MPE) (4). The most common symptom of PE is dyspnea, but there are many signs and symptoms that lead to this diagnosis. The diagnosis of PE is almost based on physical examination but paraclinical tests like CXR can help to confirm it (5). Also laboratory tests such as pleural LDH and protein and comparison with simultaneous serum level of these markers can distinguish the causes of PE (6). Treatment of PE depends on managing the underlying cause, hence it is important to diagnose it as soon as possible (5,7, 8)

Recent researches suggested that pleural D-dimer levels can be a helpful marker to propose malignancy as the cause of PE (9–11). D-dimer is a peptide that results from fibrin cleavage during homeostasis. Its serum levels increase in many physiological and pathological states such as inflammation and hypercoagulable state (12,13). It has been shown that the hypercoagulable state in MPE fluid prevents fibrin aggregation and leads to increase production of D-dimer (9).

The aim of this study was to compare serum and pleural D-dimer levels between malignant and non-malignant PE patients.

## MATERIALS AND METHODS

### Study Subjects

From April 2016 to January 2017, patients with PE who referred to the Department of Respiratory Medicine, Al-Zahra Hospital of Isfahan University of Medical Sciences for further diagnostic investigation were recruited for this study. Our study included patients between 18 and 80 years of age whose diagnosis of PE was made by radiography and clinical examination. Patients with recent history of surgery or any pre-existing coagulopathy were excluded. 64 patients with PE entered the study; thirty-two patients were diagnosed with MPE, including 8 patients with lung cancer, 7 patients with colon cancer, 6 patients with breast cancer and 4 patients with ovarian cancer and 7 with other metastatic cancer. MPE is defined as presence of malignant cells on cytological examination or in a biopsy specimen (9). Thirty-two patients with Non-Malignant Pleural Effusion (NMPE) were enrolled as control subjects, including 16 patients with transudate caused by heart failure, 7 patients with para-pneumonic effusion, 6 patients with pulmonary embolism and 3 transudates without evidence of cancer. PE was attributed to heart failure if clinical, Electrocardiographic (ECG), radiographic and echo graphic evidence of congestive heart failure was found. Pulmonary embolism was confirmed by Multidetector-row Computed Tomography Angiography (MDCTA) and also pneumonia was diagnosed based on patients’ clinical features and their chest X ray results.

### Sample Collection and D-dimer Measurement

Written consents were obtained from all the patients prior to the procedure. Thoracocenthesis was performed; pleural and simultaneous blood samples were collected and analyzed immediately. Plasma and pleural D-dimer were assayed by Enzyme-Linked Immunosorbent Assay (ELISA). Sample analysis was performed in Department of Laboratory Medicine, Al-Zahra Hospital of Isfahan University of Medical Sciences, which has met the applicable standards for accreditation. The laboratory studies were blinded to the etiology of the PE. Patients were compared in two groups with sex and age matched.

Data analysis was performed using SPSS version 18 (SPSS Inc. Chicago, IL). One-Sample Kolmogorov-Smirnov test was done to determine normality of D-dimer levels. T-test was used to evaluate D-dimer level difference between malignant and non-malignant groups. Pearson statistical test was used to determine correlation between serum D-dimer level and pleural fluid D-dimer level in each malignant or non-malignant group. P < 0.05 was considered as statistically significant.

### Ethical considerations

This study was approved by Research Ethics Committee of the Research Department, Isfahan University of Medical Sciences. Patients were completely informed about the process of the study and participated in the survey voluntarily.

## RESULTS

In this survey, 64 patients with PE were separated into two groups; 32 patients in malignant group and 32 patients in non-malignant group. Table 1 summarized the demographic and clinical characteristics of the study population. Kolmogorov-Smirnov test showed that D-dimer levels were normally distributed (P>0.05). Sex ratio and age range were the same in both groups.

**Table 1.** Clinical and demographic data of the study population (n=64).

**Age** (Mean±SD)	61.3±12
**Sex**	
Male	35
Female	29
**Diagnosis, n**	
MPE	32
Lung cancer	8
Colon cancer	7
Breast cancer	6
Ovarian cancer	4
Other metastatic cancer	7
NMPE	32
CHF	16
Pneumonia	7
PTE	6
Other^*^	3

The highest D-dimer levels (ng/dl) in non-malignant group were noted in PTE; 4183 ±696 and 8600±569 in pleural fluid and plasma, respectively.

**Figure 1.**
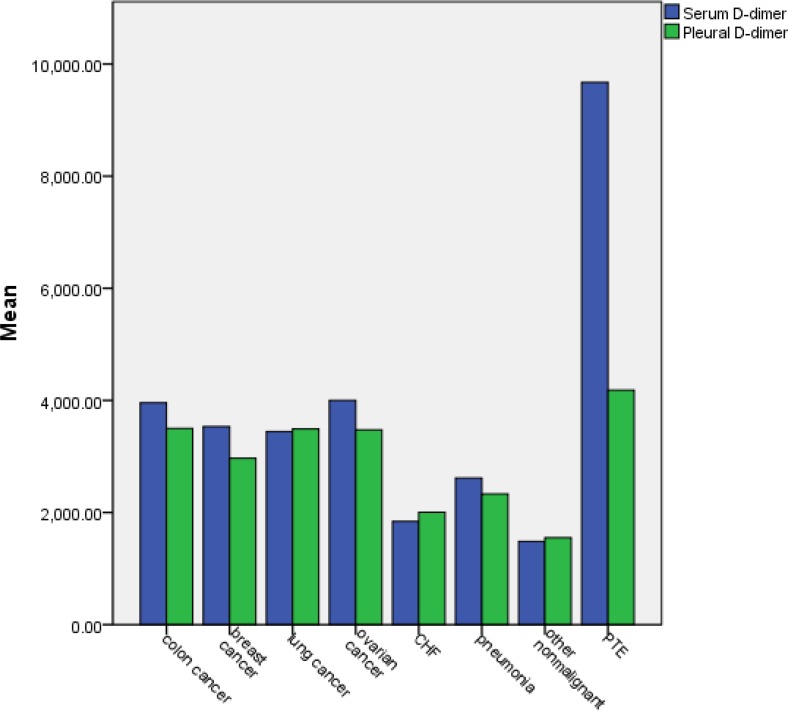
Pleural and serum levels of D-dimer according to underlying disease.

One way analysis of variance showed that there were no statistically significant differences between each disease in malignant groups according to the pleural level of D-dimer(P>0.05) and also there were no significant differences between each disease in malignant group based on serum level of D-dimer(P>0.05). Figure 1 showed serum and pleural D-dimer levels in participants according to their underlying disease.

Table 2 showed that there were statistically significant differences between malignant and non-malignant effusions according to the pleural levels of D-dimer (P=0.01). However, there were no statistically significant differences between malignant and non-malignant effusions in comparison of serum levels of D-dimer.

**Table 2.** Comparison between malignant and non-malignant effusion levels of D-dimer in serum and effusion

		**Malignant Mean ± SD**	**Non-malignant Mean ± SD**	**Mean difference**	**t test**
**Serum D Dimer (ng/ml)**	P>0.05	3472±1312	3425±3205	46	0.07
**Pleural D Dimer (ng/ml)**	P=0.01^*^	3259±1220	2425± 1311	833	2.63

*. Statistically significant at the 0.01 level.

Analysis of data with Pearson showed that there was a statistically significant correlation between pleural levels of D-dimer and serum levels of D-dimer in malignant group (P=0.01, r = 0.627) and a significant correlation was found between serum D-dimer and pleural D-dimer in non-malignant group (P = 0.001, r = 0.828).

## DISCUSSION

The diagnosis of MPE remains to be a clinical challenge and absence of feasible, reliable and minimally invasive biomarkers for MPE detection has been a limiting factor in clinical practice (6). Previous studies have assessed the role of D-dimer in pleural effusion as a feasible way to diagnose MPE (9,14).

The purpose of this study was to compare D-dimer levels in patients with benign and malignant PEs. Our results indicated that pleural levels of D-dimer increased in MPEs. The mean level of pleural D-dimer was significantly higher in MPEs than NMPEs (3259±1220 in malignant versus 2425±1311 in non-malignant) (Table 2).

MPE causes activation of the coagulation process in the pleural space, resulting in the formation of D-dimer as a split product of fibrin. Therefore, elevated levels of D-dimer are expected both in the blood and in the pleural fluid (9).

Our results coincided with those found by Dikensoy et al. (15) in their Chinese study. They reported that comparison of D-dimer levels in each group between bloody vs. non-bloody effusions showed a significant difference in only MPE group.

On the contrary of our results Philip-Joët et al. reported that there were no significant differences between malignant and non-malignant PEs regarding pleural or serum levels of D dimer; however, the number of patients was small in each disease category and therefore, their conclusions should be interpreted carefully(14). Lu et al. reported that D-dimer levels were significantly higher in pleural fluid from patients with tuberculosis pleuritis and empyema than those in pleural fluid from patients with MPE (16).

Similar to our survey, Matveychuk et al. showed high D-dimer levels among MPE and concluded that D-dimer might be useful as a simple, noninvasive, and surrogate marker for MPE (9).

Our results suggest that there was a significant correlation between pleural levels of D-dimer and serum levels of D-dimer in both malignant and non-malignant groups. Other studies showed that the coagulation system plays an important role in pleural diseases and that the understanding of pathophysiological mechanisms may open possibilities for new diagnostic and therapeutic approaches (12,17).

Our study had several limitations; first of all we enrolled only 32 patients with MPE; the limited patients’ number may affect the application of our findings. Secondly, our study is an observational study; we did not do further work on the detail mechanism on how malignancy affects local fibrinolysis system. Further studies at a large scale and aiming to investigate the detailed mechanism should be carried out to confirm our findings.

## CONCLUSION

Increasing of the level of D-dimer in pleural fluid and its relationship with MPE is important due to its different aspects. First, it can be used as an easy and inexpensive marker beside other tumor markers for better diagnosis of MPE. Second, it defines the necessity of preventive treatment of thrombosis in malignant patients. It seems that more studies are required to investigate the role of D-dimer to differentiate between MPE and other causes of pleural effusions.

It’s suggested to design a study to investigate more patients with MPE and measure more coagulation factors, so the relationship between other coagulation marker of disease and the pleural level of D-dimer can be exactly investigated. Increasing the sample size can lead to more accurate evaluation of level of D-dimer in different etiologies of PE in future researches.
